# Abnormal Distribution and Function of Circulating Monocytes and Enhanced Bacterial Translocation in Major Depressive Disorder

**DOI:** 10.3389/fpsyt.2019.00812

**Published:** 2019-11-15

**Authors:** Miguel Angel Alvarez-Mon, Ana Maria Gómez, Arancha Orozco, Guillermo Lahera, Maria Dolores Sosa, David Diaz, Enrique Auba, Agustín Albillos, Jorge Monserrat, Melchor Alvarez-Mon

**Affiliations:** ^1^Department of Psychiatry and Medical Psychology, Clinica Universidad de Navarra, Pamplona, Spain; ^2^Department of Medicine and Medical Specialties, University of Alcalá, Madrid, Spain; ^3^Institute Ramón y Cajal for Health Research (IRYCIS), Madrid, Spain; ^4^Division of Psychiatry, University Hospital “Principe de Asturias”, Alcalá de Henares, Spain; ^5^Biomedical Research Centre for Mental Health Network (CIBERSAM), Madrid, Spain; ^6^Biomedical Institute for Liver and Gut Diseases (CIBEREHD), Instituto de Salud Carlos III, Madrid, Spain; ^7^Service of Gastroenterology, University Hospital Ramón 80 y Cajal, Madrid, Spain; ^8^Immune System Diseases and Oncology, Internal Medicine Service, University Hospital “Príncipe de Asturias”, Alcalá de Henares, Spain

**Keywords:** monocyte, cytokines, bacterial translocation, gut barrier damage, systemic inflammation, major depressive disorder

## Abstract

**Introduction:** Major depressive disorder (MDD) patients experience a systemic inflammatory stage. Monocytes play an important role in innate inflammatory responses and may be modulated by bacterial translocation. Our aim was to investigate the subset distribution and function of circulating monocytes, levels of proinflammatory cytokines, gut barrier damage, and bacterial translocation in MDD patients.

**Methods:** Twenty-two MDD patients without concomitant diseases and 14 sex- and age-matched healthy controls were studied. The levels of circulating CD14^++^CD16^-^ (classical), CD14^++^CD16^++^ (intermediate) and CD14^-^CD16^++^ (nonclassical) monocytes and the intracytoplasmic tumor necrosis factor (TNF)-α, interleukin (IL)-1β, IL-6, and IL-10 expression in the presence or absence of lipopolysaccharide (LPS) stimulation were analyzed by polychromatic flow cytometry. The serum TNF-α, IL-1β, IL-6, and IL-10 levels were measured by Luminex. LPS-binding protein (LBP), intestinal fatty acid-binding protein (I-FABP), and zonulin were measured by enzyme-linked immunosorbent assay (ELISA).

**Results:** MDD patients had a significant increase in the frequency of intermediate monocytes and a significant decrease in the frequency of classical monocytes compared to those in the healthy controls. MDD patients had a significantly increased percentage of classical monocytes that expressed IL-1β, intermediate monocytes that expressed IL-1β and IL6 and nonclassical monocytes that expressed IL-1β, and decreased levels of nonclassical monocytes that expressed IL6 compared to those in the healthy controls. MDD patients had significantly increased levels of circulating TNF-α, IL-1β, LBP, and I-FABP compared to those in the healthy controls. MDD patients with high LBP levels had a significant reduction in the number of circulating monocytes compared to that in the normal-LBP MDD patients, which can be mainly ascribed to a decrease in the number of intermediate and nonclassical monocytes.

**Conclusions:** We have demonstrated that compared to the healthy controls, MDD patients show a marked alteration in circulating monocytes, with an expansion of the intermediate subset with increased frequency of IL-1β and IL-6 producing cells. These patients also exhibited a systemic proinflammatory state, which was characterized by the enhanced serum TNF-α and IL-1β levels compared to those in the healthy controls. Furthermore, MDD patients showed increased LBP and I-FABP levels compared to those in healthy controls, indicating increased bacterial translocation and gut barrier damage.

## Introduction

Major depressive disorder (MDD) is a severe psychiatric disorder that is highly prevalent worldwide (1). MDD is currently ranked as the fourth-leading cause of disability and premature death in the world, and it is expected to become the second-leading contributor to overall disease burden by 2030 (2). Despite much progress has been made in understanding MDD, the pathogenesis is still largely unknown; however, the pathogenesis of MDD may be best described as a complex interplay of social, psychological and biological factors (3). Depression is associated with a chronic, low-grade inflammatory response and the activation of cell-mediated immunity, as well as the activation of the compensatory anti-inflammatory reflex system. MDD is similarly accompanied by increased oxidative and nitrosative stress, which contribute to the neuroprogression of the disorder. Among twenty-four studies that excluded any medical comorbidities, MDD was still associated with increased interleukin (IL)-6 and tumor necrosis factor (TNF)-α. There is strong support for the general hypothesis that inflammatory cytokines are significant elements in the pathogenesis of MDD (4, 5).

Monocytes mediate essential regulatory and effector functions in innate and adaptative immune inflammatory responses (6). Circulating monocytes are phenotypically and functionally heterogeneous and are divided into three major subsets based on the expression of the lipopolysaccharide (LPS) receptor CD14 and the Fc*γ*RIII low-affinity IgG receptor CD16: classical (CD14^++^CD16^-^), intermediate (CD14^++^CD16^++^), and nonclassical (CD14^-^CD16^++^) (7, 8). Activated monocytes show relevant immunomodulatory activities, including the secretion of pivotal cytokines, such as the proinflammatory cytokines IL-6, IL-1β, and tumor necrosis factor-alpha (TNF-α) and the anti-inflammatory cytokine IL-10 (9). Monocytes have been demonstrated to be involved in the pathogenesis of several organ-specific and systemic inflammatory diseases (10).

Different mechanisms may be involved in the regulation of the activation and survival of monocytes. It has been recently shown that damage to the intestinal barrier in MDD patients increases gut permeability and favors bacterial translocation (11). The passage of bacterial products such as LPS promotes a systemic inflammatory imbalance, including monocyte compromise (12). The hepatic synthesis of LPS-binding protein is induced by LPS, and in several clinical settings, plasma LBP reﬂects long-term exposure to bacteria and their endotoxins (13, 14).

Our work focused on the study of circulating monocytes and the level of bacterial translocation and gut barrier damage in MDD patients. To avoid confounding factors, we focused our study on a homogeneous population of 22 MDD patients without clinical infections or concomitant diseases that could have potential interactions with the immune system. In parallel, we studied 14 age- and sex-matched healthy controls (HCs). We analyzed the pattern of the distribution of the classical, intermediate and nonclassical circulating monocyte subsets. We also investigated the intracytoplasmic production of TNF-α, IL-1β, IL-6, and IL-10 after LPS stimulation and the serum levels of these cytokines. We measured the serum levels of LBP, I-FABP, and zonulin to study bacterial translocation and gut barrier integrity.

## Materials And Methods

### Study Protocol

In this study, we included 22 patients with MDD from the Department of Psychiatry of the Clinica Universidad de Navarra and from the Hospital Universitario Príncipe de Asturias. The inclusion criteria included the following: (a) psychiatrist-confirmed diagnosis of MDD, single or recurrent, according to the Diagnostic and Statistical Manual of Mental Disorders criteria, Fifth Edition (DSM-V) (15) (16); (b) a minimum score of 14 points on the 17-item Hamilton Rating Scale for Depression (HRSD); and (c) age, 18–65 years. Potential subjects were excluded for the following reasons: 1) acute infection in the last three months; 2) chronic bacterial or viral infection; 3) the use of steroids or immunomodulatory drugs in the last three months; 4) an autoimmune disease; 5) a cardiovascular disease, including hypertension and ischemic heart disease; 6) a hematopoietic, lung, hepatic or renal disorder; 7) an endocrine or metabolic disease, including diabetes mellitus and hypercholesterolemia, or a body-mass index (BMI) higher than 30; 8) a history of malignancy; 9) immunodeficiency and malnutrition; 10) pregnancy or lactation; and 11) concomitant psychiatric illness, as assessed with the MINI International Neuropsychiatric Interview (17). The patients were studied in parallel with 14 sex-, age-, and BMI-matched HCs from the same epidemiological area.

This study was approved by the ethics committee of the University of Navarra and the Hospital Universitario Príncipe de Asturias. After the study procedures had been fully explained, the subjects provided written informed consent before study enrollment.

Blood samples were drawn from all subjects via standard venipuncture using an established aseptic technique. Samples were obtained from the MDD patients at the time of the clinical evaluation at the outpatient clinic. Serum samples were also included for analysis. After collection, the samples were centrifuged, and the serum was isolated, aliquoted, and stored at -80°C until analysis.

### Isolation of Peripheral Blood Mononuclear Cells

Peripheral blood mononuclear cells (PBMCs) were separated with Ficoll-Hypaque (Lymphoprep^TM^, Axis-Shield, Oslo, Norway) gradient centrifugation. The cells were then resuspended in RPMI 1640 (BioWhittaker Products, Verviers, Belgium) supplemented with 10% heat-inactivated fetal calf serum, 25 mM HEPES (BioWhittaker Products) and 1% penicillin-streptomycin (BioWhittaker Products). Cell counting was performed with conventional light microscopy using a Neubauer chamber following the criteria for trypan blue dead-cell exclusion. The viability of fresh PBMCs was evaluated by both trypan blue (light microscopy) and 7-aminoactinomycin D (7-AAD) (flow cytometry) exclusion.

### Immunophenotype Studies and Intracellular Cytokines

The proportions of monocyte subsets were determined in fresh PBMCs by seven-color polychromatic flow cytometry in a FACSAria II cytometer using FACSDiva software (Becton Dickinson, NJ, USA). To analyze the production of cytokines by PBMCs, 1 million of fresh PBMCs were cultured in ultralow attachment plates (Corning Incorporated, ME, USA) (1 ml of cells at 10^6^ cells/ml) and incubated for 4 h at 37°C with 5% CO_2_. PBMC stimulation was performed by adding LPS (10 µg/ml, Sigma-Aldrich Chemistry, Spain) and monensin (50 µg/ml, Sigma). Next, the cells were labeled with CD14-PerCP and CD16-Alexa647 (Becton Dickinson) MoAbs and the vital dye Aqua-QD565. For intracytoplasmic staining, the cells were fixed and permeabilized (Fix and Perm, Caltag Laboratories), and cytokines were stained with IL-1β-FITC, IL-10-PE, IL-6-V505 and TNF-α-Alexa700 (Becton Dickinson) MoAbs.

### Quantification of Serum Cytokines Using Luminex

To study the concentrations of IL-6, IL-1β, TNF-α, and IL-10 cytokines in the serum, the Milliplex MAP Kit (MERCK, Darmstadt, Germany) was employed using the protocol recommended by MERCK. The plate was read in a Luminex MAGPIX with xPONENT software. The concentration of each cytokine was calculated from the standard curve using mean fluorescence intensity (MFI) analysis with Analyst software (MERCK).

### Study of The Intestinal Barrier Integrity and Bacterial Translocation

To study intestinal barrier damage, an analysis of FABP and zonulin concentrations in the serum was performed by enzyme-linked immunosorbent assay (ELISA). I-FABP was purchased from Hycult Biotech (Hycult Biotech, PA, USA), and zonulin was purchased from R&D Systems (R&D Systems, MN, USA). The tests were carried out according to the protocols provided in the kits. The plate was read in an iMark Microplate Reader at 450 nm with Microplate Manager Software (Thermo Fisher Scientific, MA, USA).

To study bacterial translocation, the concentration of LBP in the serum was measured by ELISA (Abnova, Taipei, Taiwan). We performed 1:800 dilutions of the samples from the MDD patients and HCs. The test was carried out according to the protocol provided in the kit. The plate was read in an iMark Microplate Reader at 450 nm with Microplate Manager Software (Thermo Fisher Scientific).

### Statistical Analysis

Comparisons between patients and HCs were performed using a nonparametric Mann–Whitney *U* test. Associations between variables were assessed with Spearman’s rank correlation coefficient by simple linear regression analyses. All calculations were performed using the Statistical Package for the Social Sciences (SPSS, version 22.0, Chicago, IL). Significance was set at p < 0.05.

The absolute number of circulating monocyte subsets was calculated by the percentage of each subpopulation in PBMCs determined by flow cytometry multiplied by the total number of monocytes per microliter measured by Beckman Coulter, Inc (Brea, CA, USA).

## Results

### Demographic Profile of Participants

**Table 1** shows the demographic data and clinical characteristics of the 22 MDD patients and 14 HCs included in the study. No significant differences were found between MDD patients and HCs with respect to the variables that were studied, except for employment status. The patient group included 13 females and 9 males, ranging from 18 to 56 years of age. The duration of their depressive episode before entering the study was 17.16 ± 4.60 weeks. Thirteen patients (59.1%) had suffered at least one previous MDD episode. The mean value of the HRSD was 16.54 ± 1.71.

**Table 1:** Baseline characteristics.

	MDD	HC	p value
**Socio-demographic**			
Age, mean (SD)	40,3 (8.9)	40,8 (10,5)	0.44
Sex (% female)	59.1	57,1	0.91
Currently employed and active n (%)	12 (54.5)	13 (92.9)	<0.05
College degree n (%)	9 (40.9)	7 (50.0)	0.63
**Past History**			
Family history of depression n (%)	10 (45.6)	7 (50.0)	0.79
Family history of other psychiatric disorder n (%)	8 (36.4)	5 (35.7)	0.97
**Health characteristics**			
BMI, mean (SD)	26.45 (4.04)	25.26 (3.87)	0.81
Smoking n (%)			
-Never	14 (63.6)	8 (57.1)	
-Occasionally	3 (13.6)	3 (21.4)	
-Everyday	5 (22.7)	3 (21.4)	
Drinking n (%)			
-Never	7 (31.8)	3 (21.4)	
-Occasionally	12 (54.5)	10 (71.4)	
-Everyday	3 (13.6)	1 (7.1)	

All patients received pharmacological treatment at their doctor’s discretion: 2 (9.09%) were medication-free, 19 (86.36%) received antidepressant medications, 17 (77.27%) received anxiolytics or hypnotics, 5 (22.72%) received mood stabilizers, and 6 (27.27%) received atypical antipsychotics. Nineteen (86.36%) received combination pharmacotherapy, consisting of at least 2 different types of medication in 10 patients (45.45%) and of at least 3 different types of medication in the other 9 patients (40.90%). None of the patients received electroconvulsive therapy (ECT).

### MDD Patients Show an Expansion of The CD14^++^CD16^++^ Intermediate Monocyte Subset

**Figure 1:**
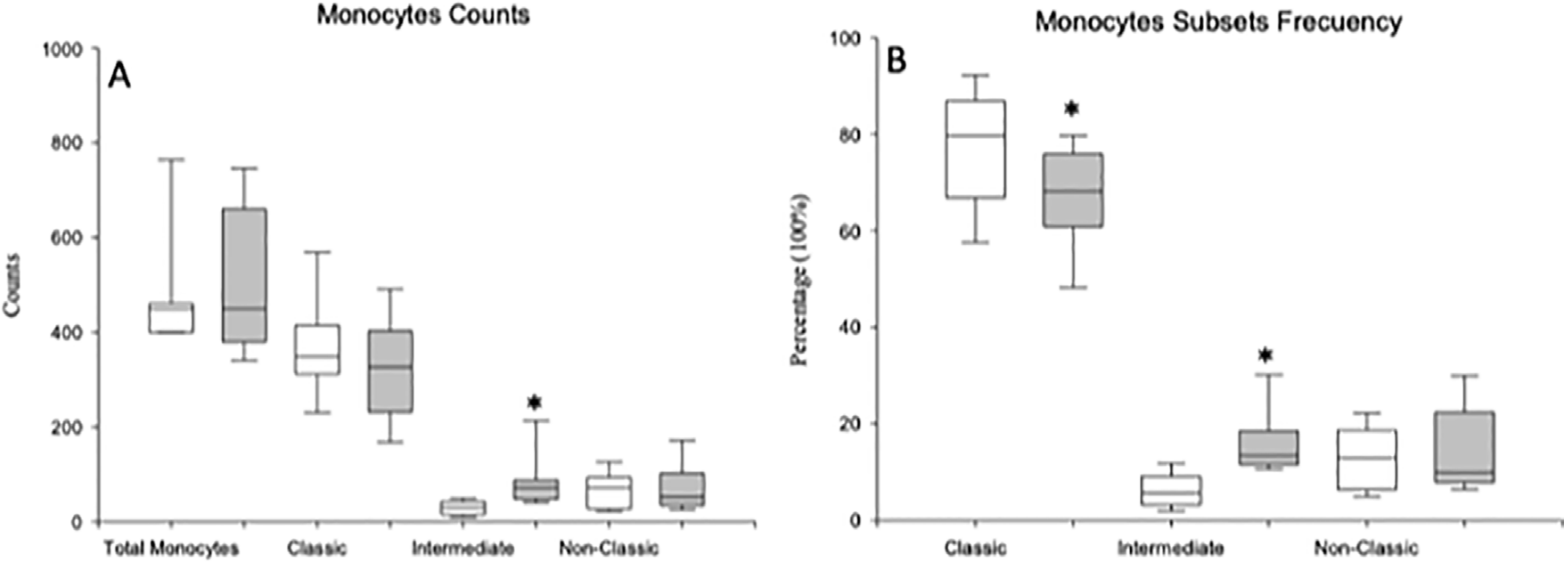
Absolute number and frequency of total circulating monocytes and of the classical, intermediate and nonclassical monocyte subsets in MDD patients and healthy controls. Absolute number (cells/µl) (y axis) of circulating monocytes and of the classical, intermediate and nonclassical subsets in MDD patients (gray box plots) and healthy controls (white box plots) are shown in panel **(A)**. Frequency of the three different monocyte subsets in the circulating monocytes in MDD patients (gray box plots) and controls (white box plots) are shown in panel **(B)**. The top of the rectangle indicates the third quartile, the horizontal line near the middle of the rectangle indicates the median, and the bottom of the rectangle indicates the first quartile. A vertical line extends from the top of the rectangle to indicate the maximum value, and another vertical line extends from the bottom of the rectangle to indicate the minimum value. *Significant difference between MDD and healthy controls for the indicated variable.

We studied the number of circulating monocytes and their subset distribution in patients with MDD and in sex- and age-matched HCs. (**Figure 1**). There were no statistically significant differences in the number of total circulating monocytes between the MDD patients and HCs. But a significant increase in the number of intermediate monocytes was found in MDD patients with respect to HCs. However, MDD patients showed a marked redistribution of the subsets of the monocyte compartment, with a significant increase in the frequency of intermediate monocytes and a significant decrease in the frequency of classical monocytes compared to those found in the HCs. There were no significant differences in the frequency of the nonclassical subset between the MDD patients and HCs.

### MDD Patients Exhibit Differential Abnormal Cytokine Production in The Monocyte Subsets

**Figure 2:**
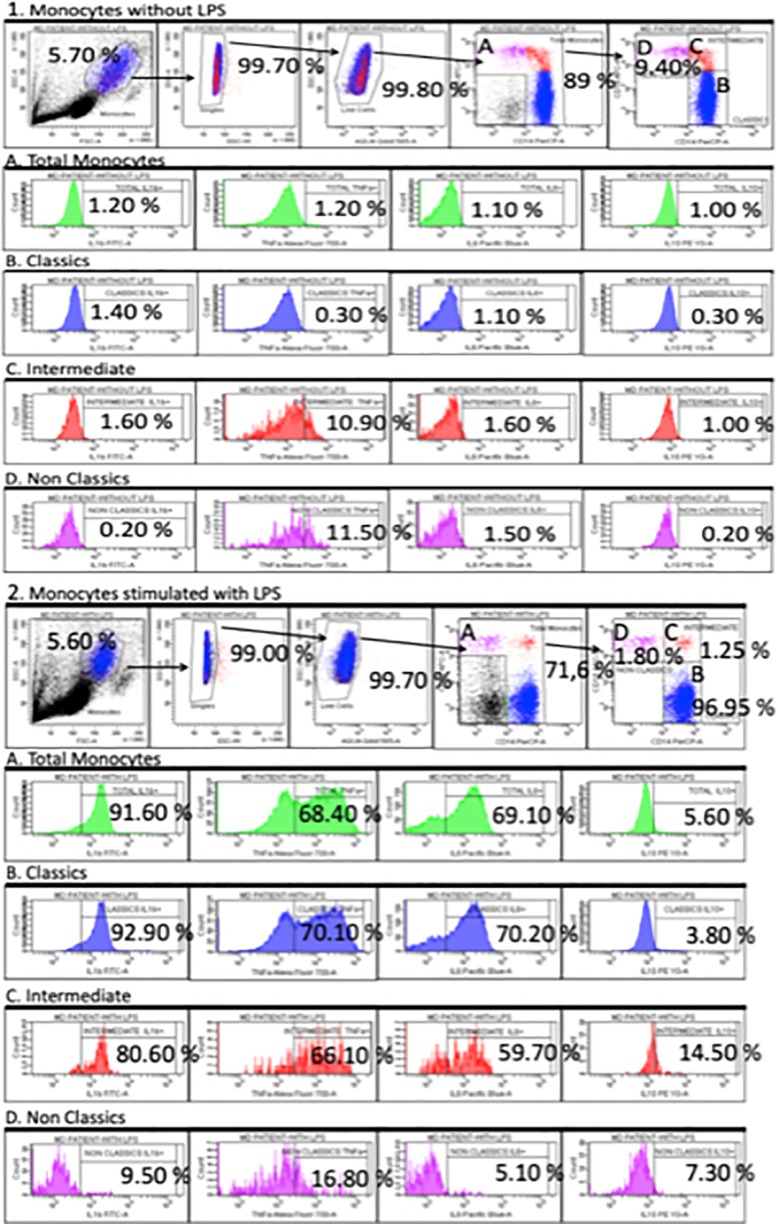
Dot plots represent the flow cytometry gating strategy and histograms of the intracellular IL-1β, TNF-α, IL-6 and IL-10 expression by total circulating monocytes and classical, intermediate and nonclassical subsets in a representative case of MDD. The first row dot plots represent the selected gates and percentages to obtain the total monocytes (panel **A**) and classical, intermediate and nonclassical monocyte subsets (panel **B**, **C** and **D**, respectively) in the presence or absence of LPS (5 ug/ml) stimulation for 4 hours (panel 1 and 2). Panel A, B, C and D. Histograms represent the percentages of TNF-α, IL-1β, IL-6 and IL-10-producing monocyte in the indicated monocytes subsets.

**Figure 3:**
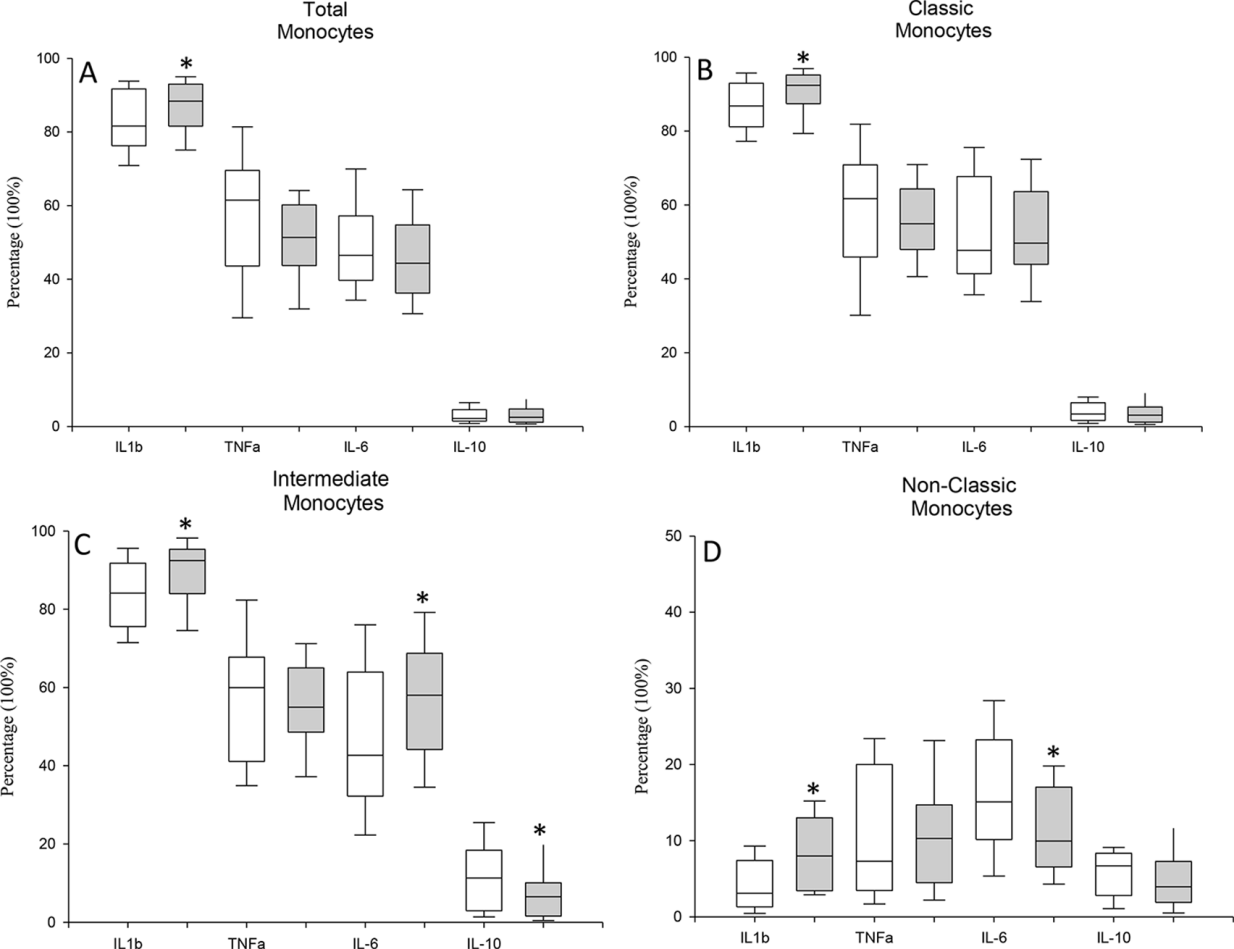
Frequency of TNF-α, IL-1β, IL-6 and IL-10 expression by circulating monocytes and classical, intermediate and nonclassical monocytes in MDD patients and healthy controls after stimulation with LPS stimulation. Frequencies of cells (y axis) in MDD patients (gray box plots) and healthy controls (white box plots) that express the indicated cytokine (x axis) after in vitro LPS (5 ug/ml) stimulation for 4 hours in total (panel **A**), classical (panel **B**), intermediate (panel **C**) and nonclassical (panel **D**) monocytes. The top of the rectangle indicates the third quartile, the horizontal line near the middle of the rectangle indicates the median, and the bottom of the rectangle indicates the first quartile. A vertical line extends from the top of the the maximum value, and another vertical line extends from the bottom of the rectangle to indicate the minimum value. *Significant difference between MDD and healthy controls for the indicated variable.

We studied the intracellular expression of TNF-α, IL-1β, IL-6, and IL-10 in the total monocyte population and in the classical, intermediate and nonclassical monocyte subsets from MDD patients and HCs after LPS stimulation. **Figure 2** shows the flow cytometry gating strategy and histograms of the intracellular IL-1β, TNF-α, IL-6, and IL-10 expression by total circulating monocytes and classic, intermediate and nonclassic subsets in a representative case of MDD. We found that the percentage of the total monocyte population that expressed IL-1β in MDD patients was significantly higher than that in healthy controls (**Figure 3**) Compared to the healthy controls, MDD patients also had a significantly increased percentages of classical monocytes that expressed IL-1β, intermediate monocytes that expressed IL-1β and IL-6, nonclassical monocytes that expressed IL-1β and decreased percentages of intermediate monocytes that express IL-10 and nonclassical monocytes that expressed IL-6.

**Figure 4:**
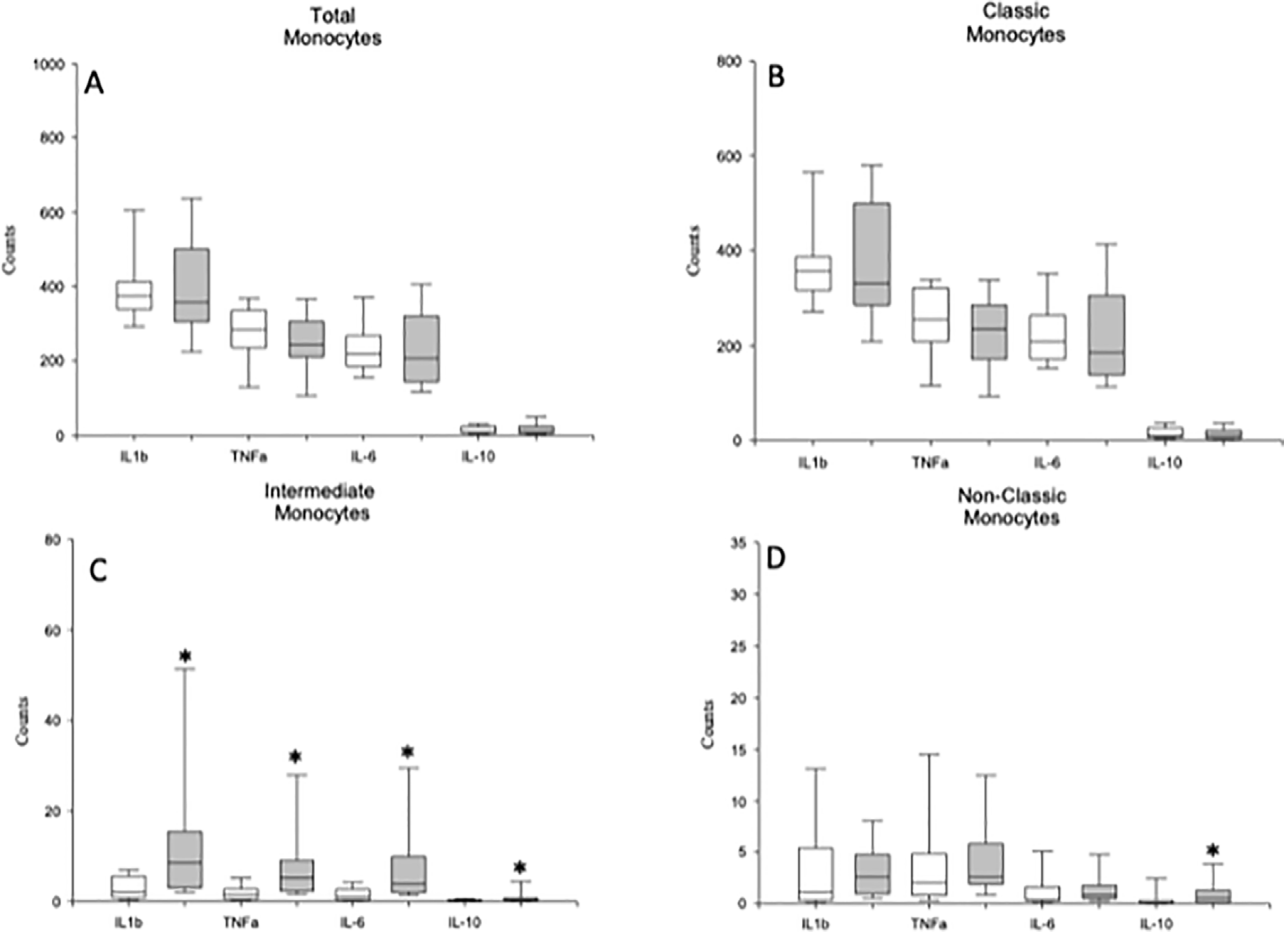
Circulating monocytes and classical, intermediate and nonclassical monocytes are able to express TNF-α, IL-1β, IL-6 and IL-10 in MDD patients and healthy controls. Absolute number (cells/µl) (y axis) of circulating monocytes (panel **A**) and of the classical (panel **B**), intermediate (panel **C**) and nonclassical (panel **D**) monocytes in MDD patients (gray box plots) and healthy controls (white box plots) that are able to express the indicated cytokine (x axis) after in vitro LPS (5 ug/ml) stimulation for 4 hours. The top of the rectangle indicates the third quartile, the horizontal line near the middle of the rectangle indicates the median, and the bottom of the rectangle indicates the first quartile. A vertical line extends from the top of the rectangle to indicate the maximum value, and another vertical line extends from the bottom of the rectangle to indicate the minimum value. *Significant difference between MDD and healthy controls for the indicated variable.

Next, in both groups of subjects, we calculated the potential number of circulating monocytes that could express TNF-α, IL-1β, IL-6, and IL-10 by multiplying the number of the different monocyte subsets by the percentage of cells that express the analyzed cytokines after LPS stimulation in the defined monocyte subsets in both groups of subjects (**Figure 4**). We observed that the number of circulating intermediate monocytes that could express TNF-α, IL-1β, IL-6, and IL-10 was significantly increased in the MDD patients compared to those found in the HCs.

**Figure 5:**
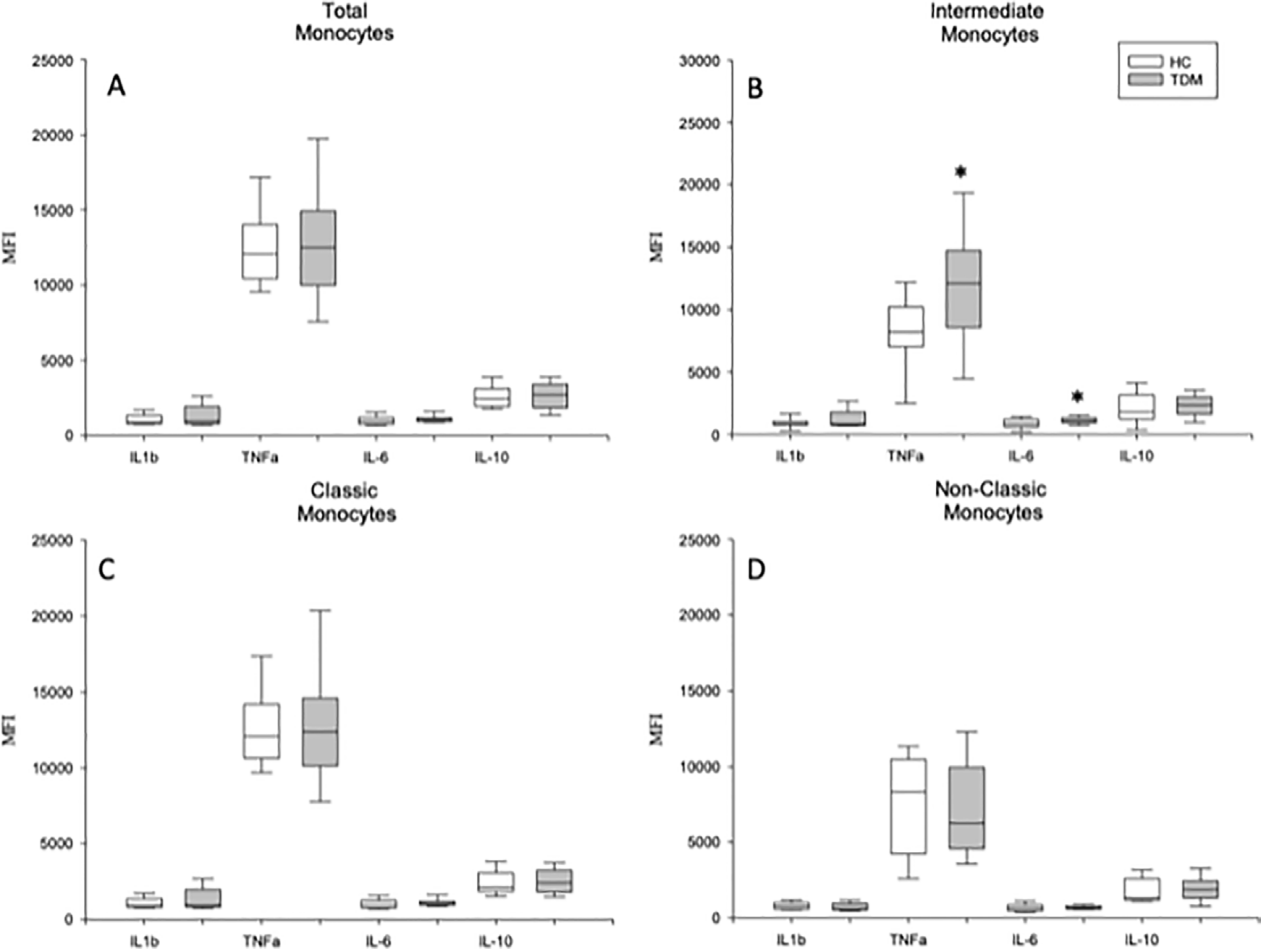
Mean fluorescence intensity (MFI) TNF-α, IL-1β, IL-6 and IL-10 expression by LPS activated circulating monocytes and classical, intermediate and nonclassical monocyte subsets in MDD patients and HCs. MFI of indicated cytokines in total monocytes (panel **A**), classic (panel **B**), intermediate (panel **C**) and non-classic monocytes subsets (panel **D** after in vitro LPS (5 ug/ml) stimulation for 4 hours from MDD patients (grey box plots) and HCs (white box plots). The top of the rectangle indicates the third quartile, a horizontal line near the middle of the rectangle indicates the median, and the bottom of the rectangle indicates the first quartile. A vertical line extends from the top of the rectangle to indicate the maximum value, and another vertical line extends from the bottom of the rectangle to indicate the minimum value. *Significant difference between MDD and healthy controls in the indicated variable.

We studied the MFI of the intracellular expression of TNF-α, IL-1β, IL-6 and IL-10 in the total monocyte population and in the classical, intermediate and nonclassical monocyte subsets from MDD patients and HCs after LPS stimulation (**Figure 5**). We found that the MFI of TNF-α and IL-6 expression on intermediate monocytes were significantly higher in the MDD patients than in HCs.

**Figure 6:**
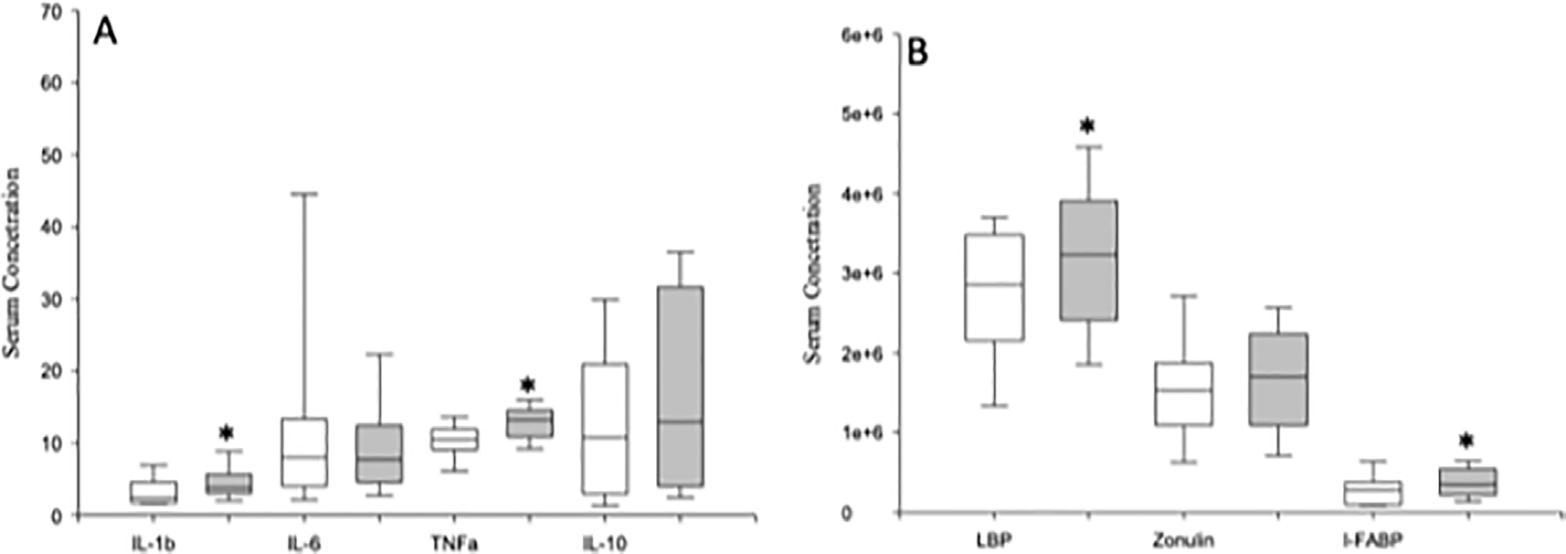
Circulating serum levels of TNF-α, IL-1β, IL-6 and IL-10 and LBP, zonulin and IFABP in MDD patients and healthy controls. Serum concentrations (pg/µl) (y axis) of TNF-α, IL-1β, IL-6 and IL-10 (panel **A**), and LBP (ng/ml), zonulin (mg/ml) and I-FABP (pg/ml) (panel **B**) in MDD patients (gray box plots) and healthy controls (white box plots). The top of the rectangle indicates the third quartile, the horizontal line near the middle of the rectangle indicates the median, and the bottom of the rectangle indicates the first quartile. A vertical line extends from the top of the rectangle to indicate the maximum value, and another vertical line extends from the bottom of the rectangle to indicate the minimum value. *Significant difference between both groups of MDD patients for the indicated variable.

We also measured the circulating levels of the proinflammatory cytokines TNF-α, IL-1β, and IL-6 and the anti-inflammatory cytokine IL-10 in MDD patients and HCs (**Figure 6**). MDD patients had significantly increased levels of circulating TNF-α and IL-1β compared to those found in HCs. We detected IL-1β, IL6, and TNF-α in the 100% and IL10 in the 91% of the serum from patients. In serum form HCs, we detected IL-1β, and TNF-α in the 100% of the samples and Il6 and IL10 in the 91% of the serums. There were no significant correlations between the serum levels and the percentages of monocytes that expressed the analyzed cytokines (data not shown).

### MDD Patients Show Increased Levels of Circulating LBP

We investigated the serum concentrations of LBP, I-FABP, and zonulin and in MDD patients and HCs (**Figure 6**). MDD patients showed significantly increased levels of LBP and I-FABP compared to those found in HCs. We detected LBP, I-FABP, and zonulin in the 100% of the serum from patients. In serum form HCs, we found zonulin in the 100% and I-FABP in the 94% of the samples. However, we only found LBP in the 50% of serums from HCs.

**Figure 7:**
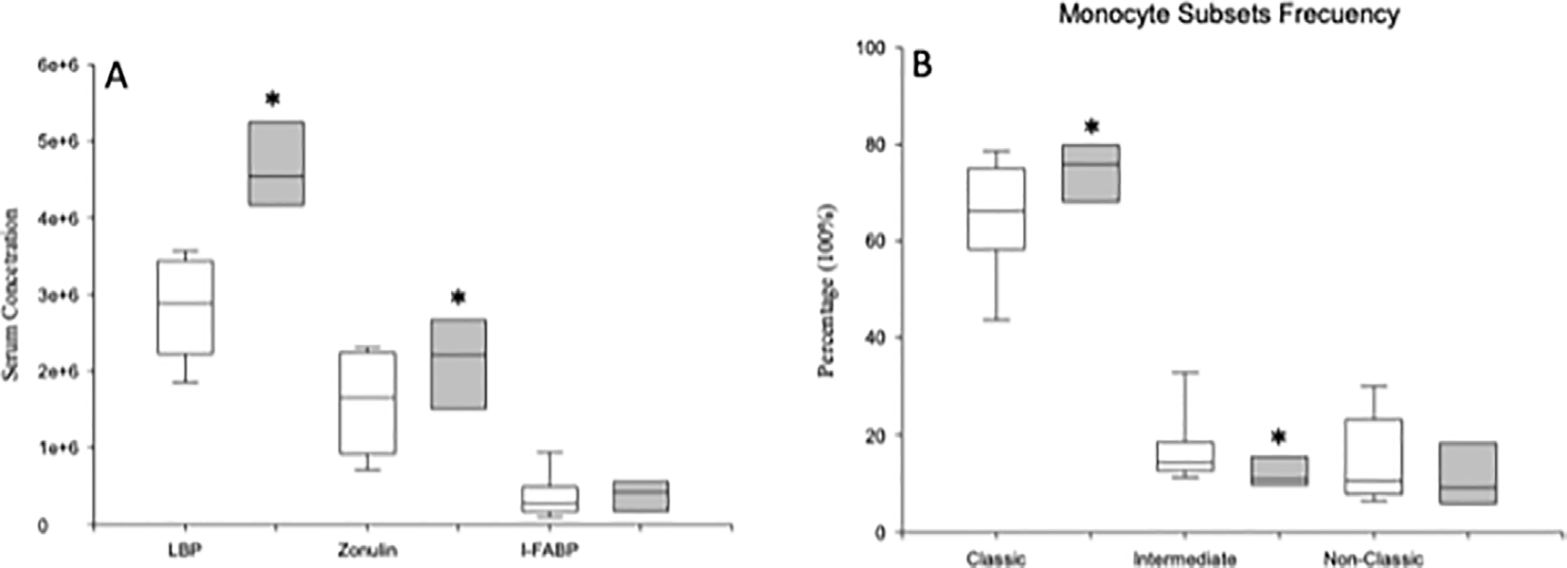
Circulating serum levels of LBP, zonulin and I-FABP and frequency of total circulating monocytes and of the classical, intermediate and nonclassical monocyte subsets in high-LBP and normal-LBP MDD patients. Serum concentrations of LBP (ng/ml), zonulin (mg/ml) and I-FABP (pg/ml) (panel **A**), and frequencies of circulating monocytes and of the classical, intermediate and nonclassical subsets (y axis) (panel **B**) in high-LBP (gray box plots) and normal-LBP (white box plots) MDD patients. The top of the rectangle indicates the third quartile, the horizontal line near the middle of the rectangle indicates the median, and the bottom of the rectangle indicates the first quartile. A vertical line extends from the top of the rectangle to indicate the maximum value, and another vertical line extends from the bottom of the rectangle to indicate the minimum value. *Significant difference between MDD and healthy controls for the indicated variable.

We found that 7 (high-LBP MDD) out of the 22 MDD patients had LBP levels higher than the 95th percentile of those found in HCs. High-LBP MDD patients had significantly increased serum zonulin concentrations compared to those with normal LBP levels (**Figure 7**). In addition, we found a significant different monocyte subset distributions in both groups of MDD patients, with a significant increase in the percentage of classical monocytes and a decrease in the intermediate monocytes in high-LBP MDD patients compared to those in normal-LBP MDD patients. However, we did not observe significant differences in the expression of TNF-α, IL-1β, IL-6, and IL-10 in the classical, intermediate and nonclassical monocyte subsets between both groups of MDD patients.

## Discussion

In this paper, we have demonstrated that compared to healthy controls, MDD patients show a marked disturbance of circulating monocytes, with the expansion of the intermediate subset with increased frequency of IL-1β and IL-6 producing cells. Concurrently, classical monocytes are decreased but have augmented frequency of IL-1β producing cells compared to those in the healthy controls. These patients also show a systemic proinflammatory state, characterized by enhanced serum TNF-α and IL-1β levels compared to those in the healthy controls. Furthermore, augmented circulating levels of LBP and I-FABP are found in MDD patients compared to those in healthy controls, indicating that these patients experience increased bacterial translocation and gut barrier damage.

The cause of MDD remains only partially defined. It is known that depression is coupled with the dysregulation of the immune-inflammatory system (18–20). The relevance of the innate immune system in the pathogenesis of MDD has been proposed (21–23). Monocytes are a cornerstone of the immune system that link innate and adaptive immunity and play critical roles in the response to bacterial infections and in the induction and regulation of the inflammatory response (6, 24). Our findings show that MDD patients have normal monocyte counts, which is consistent with previous reports (25–30). Circulating monocytes represent a versatile and dynamic cell population that is composed of different functionally heterogeneous subsets (7, 8). In MDD patients, our data demonstrate a marked redistribution of the circulating monocyte subsets, with an expansion of the intermediate monocytes and a decrease in classical monocytes compared to those of the healthy controls. These findings support the notion of the increased differentiation of classical to intermediate monocytes in MDD patients compared to that in healthy controls (24). Interestingly, the expansion of intermediate monocytes has been observed in various inflammatory diseases (31, 32). The prognostic value of the number of intermediate monocytes has also been shown for cardiovascular events in some chronic diseases (33–35). Furthermore, a reduction in classical monocytes has been previously described in chronic inflammatory diseases (36). Since the MDD patients included in our study did not suffer from inflammatory, cardiovascular or metabolic disorders, our findings support the hypothesis that the monocyte subset shifts found in MDD patients are not a consequence of comorbid somatic disorders.

Conflicting results regarding the classical and nonclassical monocyte subset frequencies have been previously described in MDD patients (25, 28, 29, 37). The differences in methodology and study populations may explain the observed discrepancies between earlier and current studies. Using a similar experimental methodology, our observations of the expansion and retraction of the intermediate monocytes and classical monocytes in MDD patients agree with recently published results (37). Furthermore, the percentage of nonclassical monocytes is normal in MDD patients. These findings give an explanation for the normal monocyte subset distribution in MDD patients with the simple analysis of two subsets, without the specific study of the intermediate and nonclassical monocytes (25, 28, 29). Differences in the somatic characteristics of the patients, such as age, BMI, the severity of the disease, and epidemiological characteristics, such as diet, might also be involved in the differences in the previous results.

In MDD patients, we also investigated the proinflammatory function of monocytes by analyzing the cellular TNF-α, IL-1β, IL-6, and IL-10 expression. Our results show that there is an abnormal pattern of proinflammatory cytokine expression by the monocyte subsets in MDD patients. The expanded intermediate monocytes show increased frequency of IL-1β and IL6 producing cells as wells as enhanced production of both cytokines. The classical and nonclassical monocytes show increased frequency of IL-1β producing cells, supporting a proinflammatory bias of these cells in MDD patients. In addition, nonclassical monocytes show decreased IL6 and TNF-α compared to those in the healthy controls. This functional heterogeneity and the different frequency of the three subsets affect the analysis of cytokine expression by circulating monocytes. Interestingly, in MDD patients, the production of IL1, IL6, and TNF by circulating immune cells has been described as being increased, normal or decreased compared to those in healthy controls (38–42). These conflicting results may be explained by the different cellular preparations, experimental models and measurement technologies employed, as well as the clinical characteristics of the MDD patients and controls who were analyzed. Our results clearly establish that the study of monocytes in MDD patients requires specific analysis at the subset level and gives some insight to aid in the understanding of the discrepancies among previous studies. The analysis of the whole monocyte population hides the quantitative and functional alterations found in the classical, intermediate and nonclassical subsets in MDD patients.

The levels of circulating cytokines and their potential relevance as clinical biomarkers have been extensively studied in MDD patients. The reported results are heterogeneous, but increases in the circulating concentrations of IL1, IL6 and/or TNF have been observed in two meta-analyses (43, 44). Consistent with previous studies, we found an increase in the inflammatory mediators IL-1β and TNF-α in MDD patients compared to those in healthy controls, but we observed normal IL-6 and IL-10 levels in MDD patients. The sources of the enhanced proinflammatory TNF-α and IL-1β levels appear to be complex. Several immune and nonimmune cells may produce IL-1β and TNF-α (45–47). It is possible to suggest that the increased frequency of IL-1β producing cells and the increased expression of this cytokine found in intermediate monocyte subset, might contribute to the increased serum levels of this cytokine found in MDD. Furthermore, the enhanced frequency of IL-1β producing cells by the classical and normal nonclassical subsets may also be involved. However, the normal TNF-α expression by the three monocyte subsets excludes these cells as being the cellular source of the increased serum levels of this cytokine that are found in MDD patients. However, these “ex vivo” results obtained after “in vitro” cellular activation may do not reflect what is happening “in vivo”. Several mechanisms may be involved in the pathogenesis of the proinflammatory monocyte abnormalities found in MDD patients. Our strategy of inclusion for the MDD patients excludes the potential pathogenic role of concomitant inflammatory, metabolic and organic diseases.

It has been shown that bacterial translocation is a cause of immune system stimulation and systemic inflammation (48). We investigated whether MDD patients suffer from bacterial translocation. The hepatic synthesis of LBP is promoted by LPS, and LPS-LBP complexes bind to CD14 on the monocyte surface. LBP peaks in the plasma 2 to 3 days after transient bacteremia or endotoxemia, and the levels remain increased up to 72 h later (49). Indeed, in several clinical settings, plasma LBP seems to better reﬂect long-term exposure to bacteria and their endotoxins than endotoxin itself (50). Our data show that as a group, MDD patients show increased LBP serum levels, in agreement with a recent observation and a previous finding of an increased plasma concentration of LPS in MDD patients (11, 51). The clinical setting described here, with increased serum LBP levels in MDD patients, differs from that of sepsis and septic patients, in which massive, acute LPS exposure promotes very high LBP concentrations (52). This finding supports the notion of the pathogenic relevance of bacterial translocation in the damage of monocytes in MDD patients (53).

Several sources may be involved as the origin of this bacterial translocation found in MDD patients. However, our inclusion criteria exclude the existence of a concomitant or recent (at least three months prior to the study) bacterial infection. The so-called gut-brain axis, linking the emotional and cognitive brain centers with gastrointestinal function, has recently received substantial attention in relation to psychiatric disorders (54–56). We have investigated gut barrier damage in MDD patients by quantifying I-FABP and zonulin, which are validated peripheral blood markers of gut function (57, 58). We found increased serum I-FABP in MDD patients compared to that in the healthy controls. This finding supports the existence of a disturbance in the intestinal barrier integrity of MDD patients. It has been reported that increased intestinal permeability favors bacterial translocation (59); increased intestinal permeability has been suggested as a pathophysiological mechanism in metabolic disorders (60), rheumatoid disorders (61, 62) and HIV infection (63), all of which are conditions with a persistent inﬂammatory component and a heightened risk of depression (64–67). Although the cross-sectional design of the current study precludes any causal inferences, it is possible to suggest that gut barrier damage might play a critical role in the increased bacterial translocation found in MDD patients. Although any causal relationships have not yet been determined, our data support the notion that gastrointestinal alterations may actually cause the low-grade systemic inflammation found in MDD patients.

## Data Availability Statement

The datasets generated for this study are available on request to the corresponding author.

## Ethics Statement

The studies involving human participants were reviewed and approved by the ethics committee of the University of Navarra and the Hospital Universitario Príncipe de Asturias. The patients/participants provided their written informed consent to participate in this study.

## Author Contributions

MAA-M: drafting the manuscript, enrolled the patients and interpretation of data; AG: execution of experiments, acquisition of data and analysis of data; AO and EA: enrolled the patients; GL: interpretation of data and revision of the manuscript for important intellectual content; MS and DD: execution of experiments; AA: revision of the manuscript for important intellectual content; JM: main supervisor of all the experiments and conducted the statistical analysis; MA-M: conception and design, obtaining funding and contributed as main supervisor of all the cited stages. All of the authors have given final approval.

## Funding

This work was partially supported by grants from the *Fondo de Investigación de la Seguridad Social*, *Instituto de Salud Carlos III* (PI18/01726), Spain, Programa de Actividades de I+D de la Comunidad de Madrid en Biomedicina (B2017/BMD-3804), Madrid, Spain and Instituto de Salud Carlos III CIBER Enfermedades hepáticas y Digestivas, Spain.

## Conflict of Interest

The authors declare that the research was conducted in the absence of any commercial or financial relationships that could be construed as a potential conflict of interest.
